# Beyond getting the numbers right: what does it mean to be a “successful” Bayesian reasoner?

**DOI:** 10.3389/fpsyg.2015.00712

**Published:** 2015-06-02

**Authors:** Gaëlle Vallée-Tourangeau, Miroslav Sirota, Marie Juanchich, Frédéric Vallée-Tourangeau

**Affiliations:** ^1^Department of Psychology, Kingston University LondonKingston upon Thames, UK; ^2^Department of Management, Kingston University LondonKingston upon Thames, UK

**Keywords:** Bayesian inference, insight problem solving, judgment and decision-making, performance, cognitive processes

Price (in Bayes, 1958) introduced Bayes's theorem as a precise and accurate method for measuring the strength of an inductive argument. He contrasted Bayesian reasoning with common sense, which, he argued, is imbued with vagueness and often erroneous. Nearly two centuries later, Price's claim was put to the test by psychologists who examined how people revise their opinions in light of new evidence (e.g., Phillips and Edwards, 1966; Kahneman and Tversky, 1973). For the past four decades, scholars have debated whether common sense can or cannot approximate Bayesian reasoning.

Contrary to Price's claim, earlier studies using a bookbags-and-poker-chips paradigm found that people did follow Bayesian prescriptions to revise judgments although their numerical answers were conservative: the psychological impact of new evidence on one's belief was less pronounced than warranted (Edwards, 1968). A paradigm shift ensued with the advent of the heuristic-and-biases programme of research (Kahneman et al., 1982). Scholars started to use vignette studies modeled after the so-called “textbook paradigm” or the “social-judgment paradigm” (Bar-Hillel, 1983). This also led to an about-turn in the portrayal of people's ability to revise their judgments accurately. Vignette studies did not showcase mere conservatism, they elicited biased judgments which were often in blatant contradiction with Bayesian prescriptions. This bleak picture of people's ability to form Bayesian judgments was once more overturned in the mid-nineties when researchers demonstrated that natural frequency formats could lead to a fourfold improvement in performance rates (Gigerenzer and Hoffrage, 1995; Cosmides and Tooby, 1996). This finding once more shifted the point of scholarly contention as scholars started to debate whether the improvement observed arises from the use of natural frequencies in and by itself or from the more effective “nested representation” that this information format elicits (Sirota et al., 2015).

Throughout this (admittedly) short history of the psychological study of Bayesian reasoning, Bayesian performance has most commonly been defined, explicitly or implicitly, as the ability to generate the “accurate” value for the posterior probability *p*(*H*|*D*), or the probability that a hypothesis *H* is true, given a new piece of evidence *D*, based on the values of *p*(*H*), *p*(not-*H*), *p*(*D*|*H*) and *p*(*D*|not-*H*) where *p*(*H*) denotes the *a priori* probability that *H* is true and *p*(not-*H*), the *a priori* probability that its alternative, not-*H* is true (which may or may not be equated with the base rates; Mandel, 2014); *p*(*D*|*H*) denotes the probability of observing *D* when we know *H* to be true; and, finally, *p*(*D*|not-*H*) denotes the probability of observing *D* when the alternative hypothesis, not-*H* is true.

This approach to performance assessment—comparing a normative numerical value to a subjective probability estimate—informs *what* is computed (a Bayesian answer, based on a correct number or a correct algorithm) and enables researchers to assess Bayesian *performance*. Efforts to improve Bayesian performance have focused on modifying environmental characteristics such as the probabilistic information format (e.g., Gigerenzer and Hoffrage, 1995). But performance arises from the coupling of the task environment and the cognitive processes applied to the task at hand. Fostering better Bayesian performance can also involve a better understanding of Bayesian *reasoning*, that is, *how* the subjective estimate is actually computed (e.g., see Sirota et al., 2014).

Adopting a reasoning-based focus also sheds light on differences between the three classic paradigms mentioned above that would otherwise remain concealed. While any of these paradigms may be used interchangeably to assess Bayesian *performance*, whether they all involve the same type of Bayesian *reasoning* is debatable. This is not a trivial distinction: if different paradigms invoke different reasoning processes, what works for improving Bayesian performance will be contingent on the particular research paradigm adopted to study performance. In the remainder of this essay, we show that a focus on performance (where participants' probability judgments are compared to Bayesian normative values or algorithms) obscures the fact there are more than one way to engage in Bayesian reasoning. Our analysis suggests three criteria against which the quality of Bayesian inferences may be assessed: an *accuracy criterion* (did participants compute the normative value? Did they apply the correct algorithm?), an *adequacy criterion* (did participants appropriately revise their initial judgment?), and a *restructuring criterion* (did participants successfully restructure their initial representation of the problem to achieve the goal state?).

The typical bookbags task involves two urns with symmetrical assortment of marbles—e.g., a “black urn” with 600 black and 400 white marbles, and a “white urn” with 400 white and 600 black marbles (Peterson et al., 1965). An experimenter selects one urn at random and hides it in an opaque box from which he then draws several samples of marbles. After observing each sample, participants are asked to revise the probability that the sample originates from one urn by moving a slider along a bar displaying 100 marks. The length of the bar's left section represents the probability that the marbles had been drawn from the black urn. Participants' output judgments can be compared with the Bayesian norm. This involves computing *p*(*D|H*) and *p*(*D*| not-*H*), the probabilities of observing the sample *D* if it were obtained from the black urn and the white urn, respectively. Even when participants are informed about the exact ratio of marbles in each urn, it is implausible to assume that they engage in such explicit numerical computations to revise their judgment. Instead, belief revision is more likely to arise from intuitive thinking processes involving an assessment of the perceptual similarity between the sample and the urn (e.g., see Read and Grushka-Cockayne, 2011). In such a context, interventions on feedback and learning from experience are more likely to improve Bayesian reasoning than manipulations of information format, for example.

Social-judgment studies of Bayesian reasoning (e.g., Kahneman and Tversky, 1973) include social scenarios and subjective probabilities implied by thumbnail verbal descriptions instead of countable numerical information. Typically, social-judgment tasks involve the assessment of the posterior probability that an individual belongs to a target category (e.g., engineer), based on both a short verbal description of the individual's social attributes (e.g., “spends most of his free time on his many hobbies which include home carpentry, sailing and mathematical puzzles” Kahneman and Tversky, 1973, p. 241) and the numerical base rate of the target category and an alternative category (e.g., 30 engineers and 70 lawyers). So while social-judgment tasks provide precise information about the base rates, the numerical values of the likelihood probabilities *p*(*D|H*) and *p*(*D*| not-*H*) of the descriptions are neither presented to, nor elicited from the participants. By comparing subjective posterior probability judgments made in this instance with judgments made for reversed base-rate distributions (e.g., 70 engineers and 30 lawyers), it is possible to evaluate the extent to which judgments are aligned with Bayesian prescriptions just as with the bookbags paradigm. Once again, however, these judgments are unlikely to arise from explicit numerical computations akin to those required to compute the Bayesian benchmark criterion since this would require that participants spontaneously generate a numerical value for *p*(*D|H*) and *p*(*D*| not-*H*). In fact, the actual origin of the estimate produced by participants in Social-judgment tasks is unclear. The attribute-substitution account (Kahneman and Frederick, 2002) theorizes that participants use a heuristic attribute (e.g., the extent to which the individual described is similar to a typical engineer) as a substitute for the target attribute (e.g., the probability that the individual is an engineer, given his description) in their assessment. This account, however, does not explain *how* people may compute the similarity index between the verbal description of an individual instance and a typical instance. Dougherty et al.'s (1999) MINERVA Decision-Making (MDM) model proposes that judgments are based on less than perfect memory retrieval of observations frequencies. The predictive value of the MDM model is established by comparing averaged simulated outputs with Bayesian computations and demonstrating that the simulations derived from the model are consistent with actual judgments observed in Social-judgment studies. This model is underpinned by two assumptions: first, that social judgments have a frequentist origin, and second that all individuals rely on the same memory-based process to compute their judgment. Both assumptions have yet to be tested empirically. In sum, more research is needed before the cognitive processes that yield such judgment methods in Bayesian reasoning can be firmly established. In this respect, representational theories of subjective probability such as Mandel's (2008) representational and assessment processes account may prove fruitful.

The last, and perhaps most prevalent, paradigm is the so-called textbook one. In this paradigm, participants are presented with explicit numerical values for all the components required for computing the posterior probability *p*(*H|D*), namely *p*(*H*)*, p*(*D|H*) and *p*(*D*| not-*H*) as in, for example, the mammography problem (Gigerenzer and Hoffrage, 1995). Once again, performance may be assessed in the same way it is assessed in bookbags tasks or in social judgment tasks: by comparing participants' judgment to the Bayesian criterion. The reasoning processes, which lead to the final judgment, however, are unlikely to be based on assessments of perceptual similarities (as in bookbags tasks) or memory retrieval of observed frequencies (as in social judgment tasks). Instead, textbook tasks require participants to reach a goal state (the posterior probability value) based on an initial state presenting the values of the base rate, hit rate and false alarm probabilities. In other words, textbook tasks require participants to apply operators to move from an initial state (the problem presentation) to a series of different states until the final goal state is reached. These tasks do not require an intuitive judgment of a probability value, they require analysis and problem-solving skills. As such, problem-solving theory can shed new light on the processes that underpin Bayesian reasoning in textbook problems.

Problem-solving theorists often distinguish between routine and non-routine problems (e.g., see Mayer, 1995). Routine problems involve the application of a known procedure to be solved. For example, 2 + 2 is a routine problem for anyone who has been taught a procedure for adding single digits. Applying the known procedure involves reproductive thinking; once the procedure is known, problem solvers can apply it again to solve similar problems. By contrast, when problem-solvers face non-routine problems, they do not possess a pre-existing solution procedure; they must engage in productive thinking and generate a novel solution to reach the goal state. Textbook problems presented to naive participants, that is participants who have not learnt to apply the Bayesian procedure to compute *p*(*H|D*), are difficult non-routine problems. Problem solvers may have some operators which they can apply (like adding values or multiplying them) but they have no means to gauge their progress or assess the validity of their final answer. This suggests that a possible way forward to better understand how participants may succeed in textbook tasks would be to consider those tasks as insight problems. From a set theoretic perspective, the prior probability *p*(*H*) corresponds to the proportion of the sample space S that is occupied by *H*. The occurrence of the outcome *d* reduces the sample space to the event *D* because the elements outside *D* are no longer possible outcomes. Consequently, the probability of *H given D* is the probability of *H* given the reduced sample space *D*. This analysis suggests that Bayesian performance in textbook problems demands that reasoners restructure their initial representation from the sample space S defined by the union of subsets *H* and *not-H* that both include *d* elements to the subset *D* that includes *h* and *not-h* elements.

To sum up, in this essay, we argued for a distinction between Bayesian performance and Bayesian reasoning. Whereas Bayesian performance can be assessed through a variety of paradigms, a focus on performance obscures the fact there are more than one way to engage in Bayesian reasoning: people may reason appropriately but perform poorly, thus committing what is known as an “error of application” (Kahneman and Tversky, 1982). Conversely, they may adopt an inappropriate line of reasoning (thus committing an “error of comprehension,” Kahneman and Tversky, 1982) but nevertheless produce an accurate judgment. Our analysis suggests three criteria against which the quality of Bayesian inferences may be assessed: an accuracy criterion, an adequacy criterion, and a restructuring criterion. Whereas the accuracy criterion is applicable in all three paradigms, the adequacy criterion is better suited to bookbags tasks because they require participants to revise an initial judgment or the social-judgment tasks because they ask participants to provide a subjective estimate that weighs numerical-explicit and subjective-implicit information. Likewise, the restructuring criterion is better suited to textbook tasks as these tasks require participants to navigate through a problem space. Each criterion also points to different strategies for improving the quality of Bayesian inferences. The accuracy criterion favors analytical accounts where reasoning is defined as the step-by-step transformation of explicit numerical quantities and facilitation results from easing the cognitive cost of carrying out these computations. The adequacy criterion favors associative accounts where reasoning is defined as belief updating and facilitation results from the better calibration of the subjective weights attributed to different inputs. Finally, the restructuring criterion favors representational accounts where reasoning is defined as navigating through a problem space and facilitation results from the clarification of the representational structure of the problem. In other words, better understanding how people arrive at their answers in the different paradigms may prove a fruitful way forward to uncover the keys to further improve the quality of naive Bayesian inferences.

## Conflict of interest statement

The authors declare that the research was conducted in the absence of any commercial or financial relationships that could be construed as a potential conflict of interest.
